# The mitral regurgitation effects of cardiac structure and function in left ventricular noncompaction

**DOI:** 10.1038/s41598-021-84233-6

**Published:** 2021-02-25

**Authors:** Qing Zou, Rong Xu, Xiao Li, Hua-yan Xu, Zhi-gang Yang, Yi-ning Wang, Hai-ming Fan, Ying-kun Guo

**Affiliations:** 1 Department of Radiology, Key Laboratory of Birth Defects and Related Diseases of Women and Children of Ministry of Education, West China Second University Hospital, Sichuan University, 20# Section 3 South Renmin Road, Chengdu, 610041 China; 2 Department of Radiology, Deyang People’s Hospital, 173# Section 3 Tai Shan Road, Deyang, 618400 Sichuan China; 3 Department of Radiology, Peking Union Medical College Hospital, Chinese Academy of Medical Sciences, Peking Union Medical College, No. 1 Shuaifuyuan, Dongcheng District, Beijing, 100730 China; 4 Department of Radiology, West China Hospital, Sichuan University, 37# Guo Xue Xiang, Chengdu, 610041 Sichuan China; 5 Key Laboratory of Resource Biology and Biotechnology in Western China, Ministry of Education, School of Medicine, Northwest University, Xi’an, 710069 China

**Keywords:** Cardiovascular diseases, Magnetic resonance imaging

## Abstract

This study evaluated the effects of mitral regurgitation (MR) on cardiac structure and function in left ventricular noncompaction (LVNC) patients. The clinical and cardiovascular magnetic resonance (CMR) data for 182 patients with noncompaction or hypertrabeculation from three institutes were retrospectively included. We analyzed the difference in left ventricular geometry, cardiac function between LVNC patients with and without MR. The results showed that patients with MR had a worse New York Heart Association (NYHA) class and a higher incidence of arrhythmia (P < 0.05). MR occurred in 48.2% of LVNC patients. Compared to LVNC patients without MR, the two-dimensional sphericity index, maximum/minimum end-diastolic ratio and longitudinal shortening in LVNC patients with MR were lower (P < 0.05), and the peak longitudinal strain (PLS) of the global and segmental myocardium were obviously reduced (P < 0.05). No significant difference was found in strain in LVNC patients with different degree of MR; end diastolic volume, end systolic volume, and global PLS were statistically associated with MR and NYHA class (P < 0.05), but the non-compacted to compacted myocardium ratio had no significant correlation with them. In conclusion, the presence of MR is common in LVNC patients. LVNC patients with MR feature more severe morphological and functional changes. Hypertrabeculation is not an important factor affecting structure and function at the heart failure stage.

## Introduction

Left ventricle noncompaction (LVNC) is a cardiomyopathy characterized by excessive trabecularization and deep intertrabecular recesses^1^. The phenotype is uncommon in cardiomyopathy and presents in isolation or associated with other heart diseases, even acquired LVNC^2^. The clinical manifestations are also complex and varied; in some cases, LVNC shows a malignant tendency and can lead to a range of serious outcomes, such as arrhythmia, heart failure, thromboembolism, and sudden cardiac death^3^. Although its diagnosis has primarily focused on the identification of trabeculation, other features are important to classify the specific subtypes of LVNC^4,5^. It is particularly important to accurately identify LVNC with potential malignancy and improve the sensitivity of the diagnosis from a clinical perspective. Mitral regurgitation (MR) is a common occurrence in the population with left ventricular dysfunction, and associated with a worse prognosis^6,7^. However, the incidence of MR and the changes in cardiac function in LVNC patients have not been fully elucidated. In addition, it is considered difficult to estimate prognosis and to identify surgical indications in LVNC patients with MR because of the complex changes in hemodynamics and highly variable clinical presentation. Therefore, the aim of the present study was to evaluate the cardiac structure and function in LVNC patients with MR and to determine their relation to clinical status and left ventricular (LV) remodeling.

## Methods and materials

The Ethics Committee of Clinical Trials and Biomedicine at the West China Hospital of the Sichuan University approved this study and the ethics committee of other author’s hospital approved this study. We confirmed that this study was performed in accordance with the Declaration of Helsinki (2000). Informed consent was obtained from all participants prior to study participation.

### Patient population

**Figure 1:**
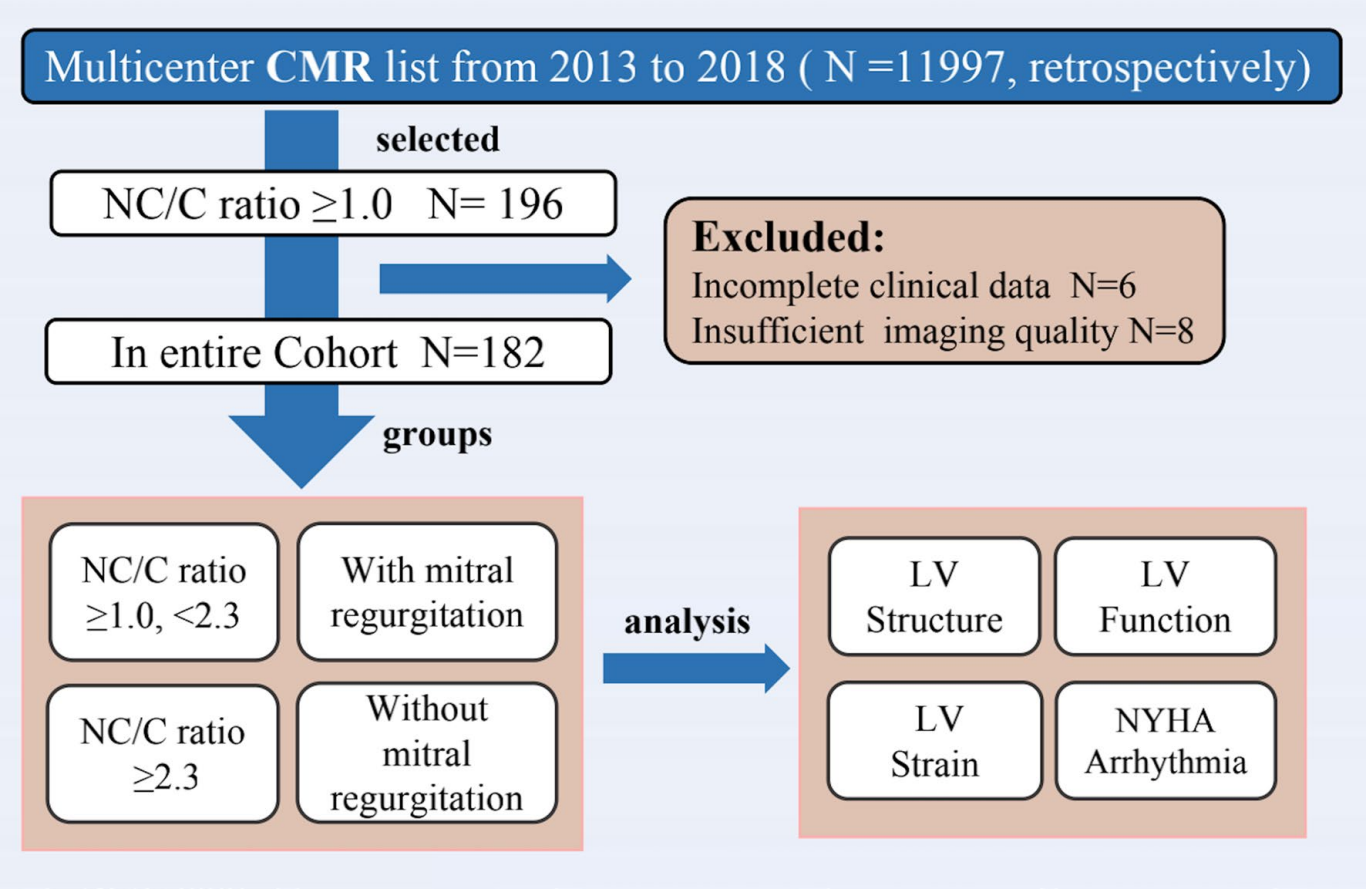
The flowchart of patient selection and implementation for the study. *CMR* cardiovascular magnetic resonance, *NC/C* noncompaction/compacted, *LV* left ventricular, *NYHA* New York Heart Association.

We queried the clinical and cardiovascular magnetic resonance (CMR) databases at three institutes for patients diagnosed with cardiomyopathy between January 2013 and December 2018. Of a total of 11,997 patients who underwent the CMR test, we extracted consecutive patients who had CMR reports that included descriptions of noncompaction or hypertrabeculation. The inclusion criteria followed Petersen et al.’s CMR criteria^8^: (1) CMR images with a distinct two-layered appearance of trabeculated and compacted myocardium; (2) subjects with increased LV trabeculation as measured by a noncompaction/compacted (NC/C) ratio ≥ 1.0 anywhere in the myocardial segments on the CMR images. Study subjects were excluded due to poor image quality (n = 6) and incomplete clinical data (n = 8), and 182 participants remained eligible (Fig. 1).

### Clinical data acquisition

The New York Heart Association (NYHA) grade and clinical symptoms, including chest pain, dyspnea and syncope/presyncope, were recorded. For resting electrocardiogram or 24-h Holter monitoring, any abnormalities, such as right or left bundle branch blocks, atrial fibrillation, and ventricular arrhythmia were summarized.

### CMR acquisition protocol

All participants in three centers were imaged on a 3.0 T magnetic resonance scanner (Magnetom Skyra, Siemens Healthcare, Erlangen, Germany) with respiratory gating technology and electrocardiogram triggering. A standard scanning protocol was used according to published international guidelines^9^, and all CMR programs and post-processing of images were standardized to minimize variation between centers. The balanced steady-state free-precession (bSSFP) sequence cine were performed in the conventional cardiac short-axis and long-axis planes (including two-chamber, three-chamber, and four-chamber images). The scanning ranges of the short axis were acquired to cover the entire LV and right ventricle.

### Assessment of mitral regurgitation by CMR

**Figure 2:**
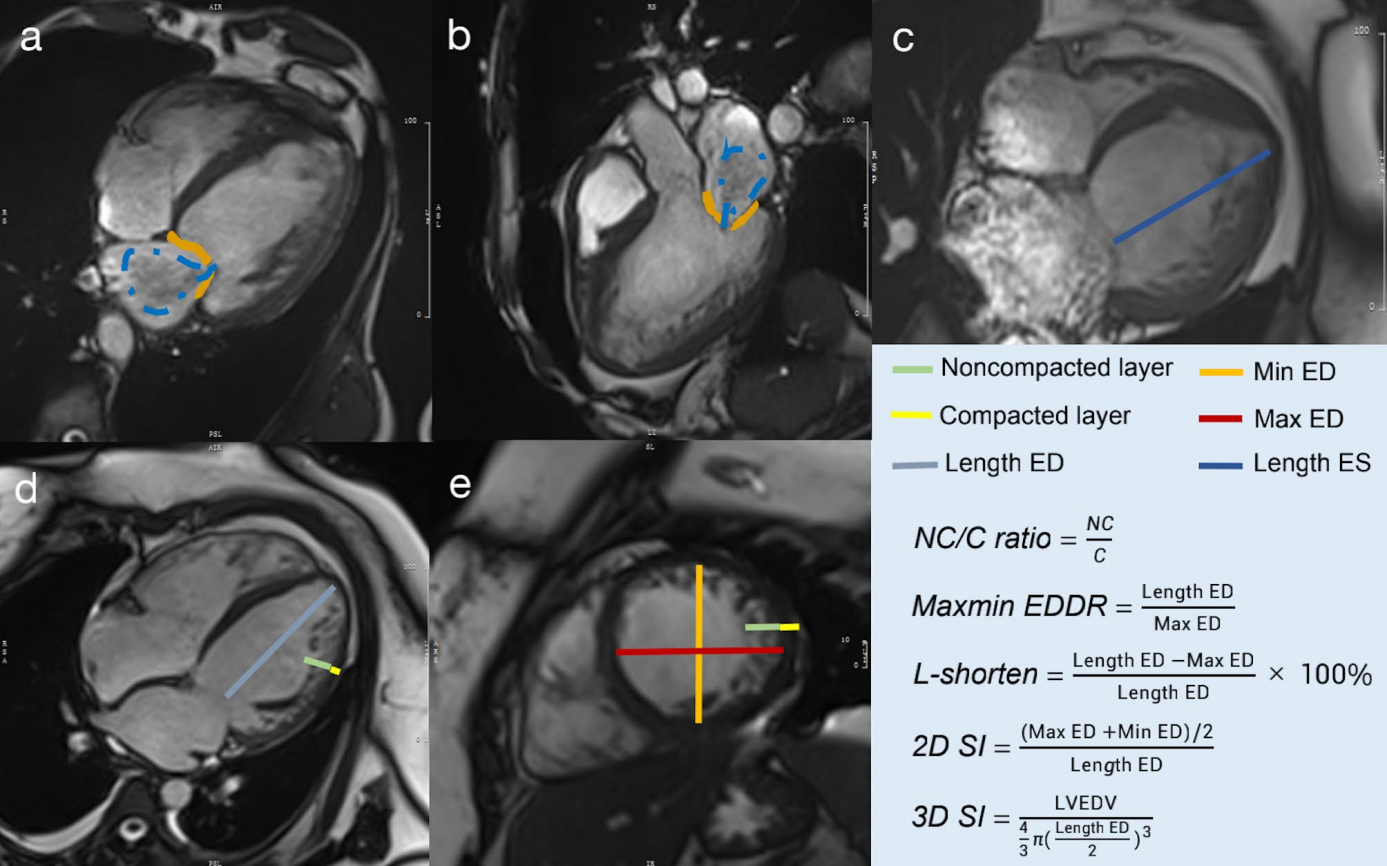
Representative images of mitral regurgitation and measurements of left ventricular geometry. **(a,b)** The yellow indicates mitral valve position, and the blue indicates blood flow through the mitral valve to the left atrium. **(c–e)** Measurements of left ventricular geometry in four-chamber cine at end-systole **(c)**, four chamber cine **(d)** and short axis cine **(e)** in end-diastole. *ED* end-diastole, *ES* end-systole, *NC/C* noncompaction/compacted, *MaxMin EDDR* maximum/minimum end-diastolic ratio, *2D SI* two-dimensional sphericity index, *3D SI* three-dimensional sphericity index, *LVEDV* left ventricular end-diastolic volume.

The assessment of mitral regurgitation and image analysis were performed by a radiologist with five years of experience in cardiac magnetic resonance during the second day after scanning. MR was diagnosed when signal voids extending from the mitral valve plane to the left atrial blood pool were detected during systole (Fig. 2a,b), according to the size and duration of mitral regurgitation, the degree of mitral regurgitation was classified into three grades: mild, moderate and severe. Mild regurgitation was defined as the presence of signal voids in one-third of systole, moderate regurgitation was defined as the duration of signal voids was more than one-third of but not throughout the whole systole, severe regurgitation was defined as signal voids throughout the whole systole^10^. In addition, all hypertrabeculation (NC/C ratio 1.0–2.3) and LVNC (NC/C ratio ≥ 2.3) patients were divided into two groups: a mitral regurgitation-positive group and a mitral regurgitation-negative group, according to CMR imaging.

### Structure and function measurements

CMR image analysis was performed using commercially available software (cvi42, Circle Cardiovascular Imaging, v. 5.6, Calgary, AB, Canada). For the cine imaging analysis, left ventricular structure and function parameters were measured on the short and long axis at the end-diastole and end-systole phases, respectively. The left ventricular geometric parameters included the ratio of noncompacted to compacted myocardium, the maximum/minimum end-diastolic ratio (MaxMin EDDR), LV longitudinal shortening (L-shorten), the two-dimensional sphericity index (2D SI) and the three-dimensional sphericity index (3D SI)^11–13^. The specific method is shown in Fig. 2c–e. Cardiac function measurements, including the LV end-diastolic volume (EDV), LV end-systolic volume (ESV), LV ejection fraction (EF), LV stroke volume (SV), and LV mass, were analyzed by manually tracing the endocardial and epicardial contours. In addition, strain analysis was performed using the release version of the same post processing software based on CMR feature tracking. The movement of the LV myocardium was quantified by automatically tracking the displacement of the endocardium and epicardium. The global and segmental tissue tracking variables included circumferential, radial, and longitudinal peak strain. Global strain is the average peak segmental strain of the entire LV except for the apical segment (17th segment) and refers to the degree of myocardial tissue deformation relative to its original length. Segmental myocardial strain is the average peak segmental strain of the apex, middle and base segments according to a 16-segment bull’s-eye diagram.

### Statistics analysis

Statistical analysis was performed using SPSS software for Windows (version 22.0, SPSS, Chicago, IL, USA). Continuous variables are expressed as the mean ± SD, and categorical data are presented as percentages and/or numbers. Continuous variables were compared using unpaired Student’s t tests or ANOVA by group (Bonferroni test), and categorical variables were compared using Fisher’s exact test or the Mann–Whitney nonparametric test if they were not normally distributed. Correlation analysis was completed by Pearson or Spearman correlation. All tests were 2-sided, and a P value of < 0.05 was considered statistically significant.

## Results

### Study population

There were 182 participants (97 men; average age 45.5 ± 10.4 years) enrolled in this study. Of all the patients, the incidence of MR in LVNC patients was higher than that in the hypertrabeculation group (n = 40, 48.2% vs n = 46, 46.5%, P > 0.05). Patients with and without MR had similar baseline ages and histories of hypertension and diabetes (P > 0.05). NYHA class ≥ III (n = 73, 40.1%) and arrhythmias (n = 96, 52.7%) were common in all participants, and patients with MR tended to have a poor NYHA functional and a high incidence of arrhythmia (P < 0.05). The arrhythmias mainly included atrial fibrillation, bundle branch blocks, and ventricular arrhythmia. Most of the patients were taking diuretics (n = 110), 83 patients were taking metoprolol, 52 were taking angiotensin receptor blockers (ARB), and 23 were undergoing angiotensin converting enzyme inhibitors (ACEI) therapy. Their clinical characteristics are summarized in Table 1.

**Table 1:** The clinical characteristics of patients.

	Overall (N = 182)	MR^−^ (N = 96)	MR^+^ (N = 86)
**Clinical**			
Age, years	45.5 ± 10.4	41.41 ± 9.57	49.46 ± 9.98
Male	97(53.3%)	49(51.0%)	48(55.8%)
Hypertension	23(12.6%)	14(14.5%)	9(10.5%)
Diabetes	19(10.4%)	11(11.4%)	8(9.37%)
NYHA class ≥ III	73(40.1%)	24(25%)	49(57.0%)*
**Arrhythmias**	96(52.7%)	43(44.8%)	53(61.6%)*
Atrial fibrillation	15(8.2%)	8(8.3%)	7(8.1%)
Bundle branch block	29(15.9%)	13(13.5%)	16(18.6%)
Ventricular contraction	52(28.6%)	22(22.9%)	30(34.9%)*
**Therapy**			
ACEI	52(28.6%)	29(28.1%)	23(26.7%)
ARB	23(12.6%)	14(14.6%)	9(10.5%)
Metoprolol	83(45.6%)	35(36.5%)	48(55.8%) *
Diuretic	110(60.4)	47(49.0%)	63(73.3%) *

*The statistical difference between MR^−^ and MR^+^ group.

*MR*^***−***^ patients without mitral regurgitation, *MR*^*+*^ patients with mitral regurgitation, *NYHA* New York Heart Association, *ACEI* angiotensin converting enzyme inhibitors, *ARB* angiotensin receptor blockers.

### Differences in LV structure and function in LVNC patients with and without MR

**Figure 3:**
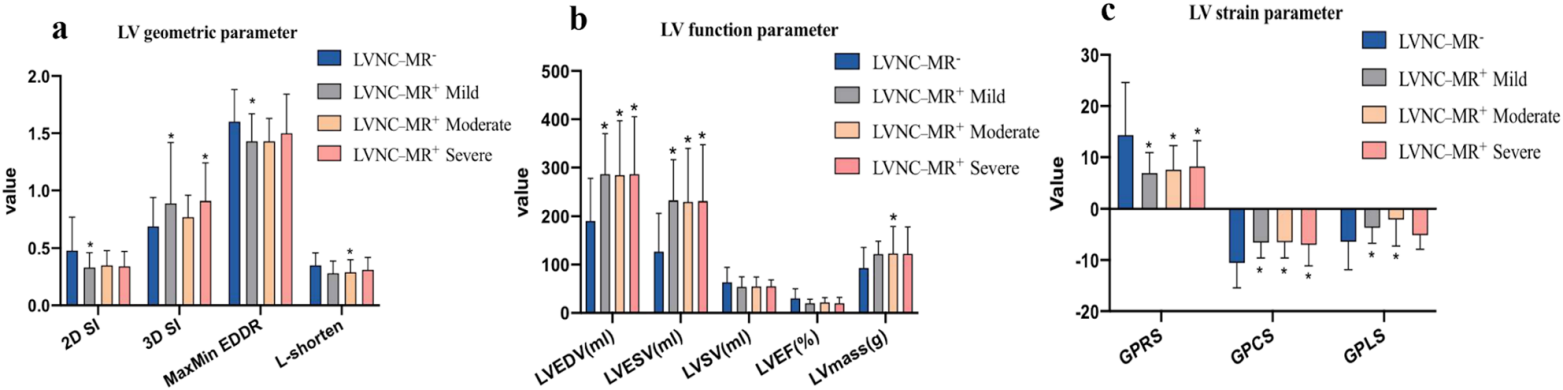
Comparison of left ventricular morphology and function in LVNC patients with different severity of mitral regurgitation. *, *P* < 0.05 (vs LVNC-MR^−^); *LVNC-MR*^*−*^ LVNC patients without mitral regurgitation, *LVNC-MR*^*+*^ LVNC patients with mitral regurgitation, *LVNC* left ventricular noncompaction, *MR* mitral regurgitation, *LVEDV* left ventricular end-diastolic volume, *LVESV* left ventricular end-systolic volume, *LVSV* left ventricular stroke volume, *LVEF* left ventricular ejection fraction, *GPRS* global peak radial strain, *GPCS* global peak circumferential strain, *GPLS* global peak longitudinal strain.

CMR findings are shown by subgroup in Table 2. In all LVNC patients, the 2D SI, MaxMin EDDR and L-shorten of patients with MR were significantly lower than those of patients without MR (2D SI: 0.34 ± 0.12 vs 0.54 ± 0.39; MaxMin EDDR:1.37 ± 0.22 vs 1.58 ± 0.31; L-shorten: 0.26 ± 0.11 vs 0.35 ± 0.12, all P < 0.05, respectively). In terms of cardiac function, the EF in LVNC patients with MR was lower than those of patients without MR (20.82 ± 9.86 vs 30.36 ± 19.96, P < 0.05), but there was no significant difference among hypertrabeculation groups. The LVEDV and LVESV in LNVC patients with MR were significantly higher than those without MR (285.73 ± 102.52 vs 190.10 ± 87.50, P < 0.05), similar results also occurred in hypertrabeculation groups (287.59 ± 101.94 vs 223.47 ± 91.02, P < 0.05), but the LVEDV and LVESV had no significant difference between LVNC and hypertrabeculation patients. There was no significant difference in LV geometric and function in LVNC patients with different degree of MR (Fig. 3a,b).

**Table 2:** The cardiac structure and function.

	Hyper-MR^−^ N = 53	Hyper-MR^+^ N = 46	LVNC-MR^−^ N = 43	LVNC-MR^+^ N = 40
**Structure**				
2D SI	0.44 ± 0.17	0.34 ± 0.13	0.54 ± 0.39	0.34 ± 0.12^#^
3D SI	0.65 ± 0.19	0.83 ± 0.48*	0.74 ± 0.29	0.88 ± 0.25
MaxMin EDDR	1.62 ± 0.26	1.52 ± 0.28	1.58 ± 0.31	1.37 ± 0.22^#^
L-Shorten	0.37 ± 0.10	0.32 ± 0.10	0.35 ± 0.12	0.26 ± 0.11^#&^
NC/C ratio	1.56 ± 0.38	1.76 ± 0.36	3.65 ± 1.92*	2.49 ± 1.46^&^
Number of NC	4.35 ± 1.65	4.73 ± 1.14	5.98 ± 1.75*	5.50 ± 1.60
**Function**				
LVEF (%)	29.99 ± 15.06	22.37 ± 13.32	30.36 ± 19.96	20.82 ± 9.86^#^
LVEDV (ml)	223.47 ± 91.02	287.59 ± 101.94*	190.10 ± 87.50	285.73 ± 102.52^#^
LVESV (ml)	159.51 ± 90.65	229.54 ± 79.47*	126.72 ± 79.48	231.04 ± 101.01^#^
LVmass (g)	31.48 ± 6.30	34.99 ± 11.45	41.28 ± 14.70*	44.50 ± 15.10^#^
LVSV (ml)	63.95 ± 21.44	58.05 ± 20.95	63.38 ± 31.13	54.60 ± 17.83
Mass/EDV	0.56 ± 0.16	0.53 ± 0.20	0.58 ± 0.41	0.45 ± 0.13

*vs Hyper-MR^−^, ^&^vs Hyper-MR^+^, ^#^vs LVNC-MR^−^.

*Hyper-MR*^*−*^ hypertrabeculation patients without mitral regurgitation, Hyper-MR^+^ hypertrabeculation patients with mitral regurgitation, *LVNC-MR*^*−*^ LVNC patients without mitral regurgitation, *LVNC-MR*^*+*^ LVNC patients with mitral regurgitation, *LVNC* left ventricle non-compaction, *2D SI* two-dimensional sphericity index, *3D SI* three-dimensional sphericity index, *MaxMin EDDR* maximum/minimum end-diastolic ratio, *L-shorten* longitudinal shortening, *NC/C* non-compacted to compacted myocardium, *LVEF* left ventricular ejection fraction, *LVEDV* left ventricular end-diastolic volume, *LVESV* left ventricular end-systolic volume, *LVSV* left ventricular stroke volume.

### Differences in the strain analysis in LVNC patients with and without MR

**Figure 4:**
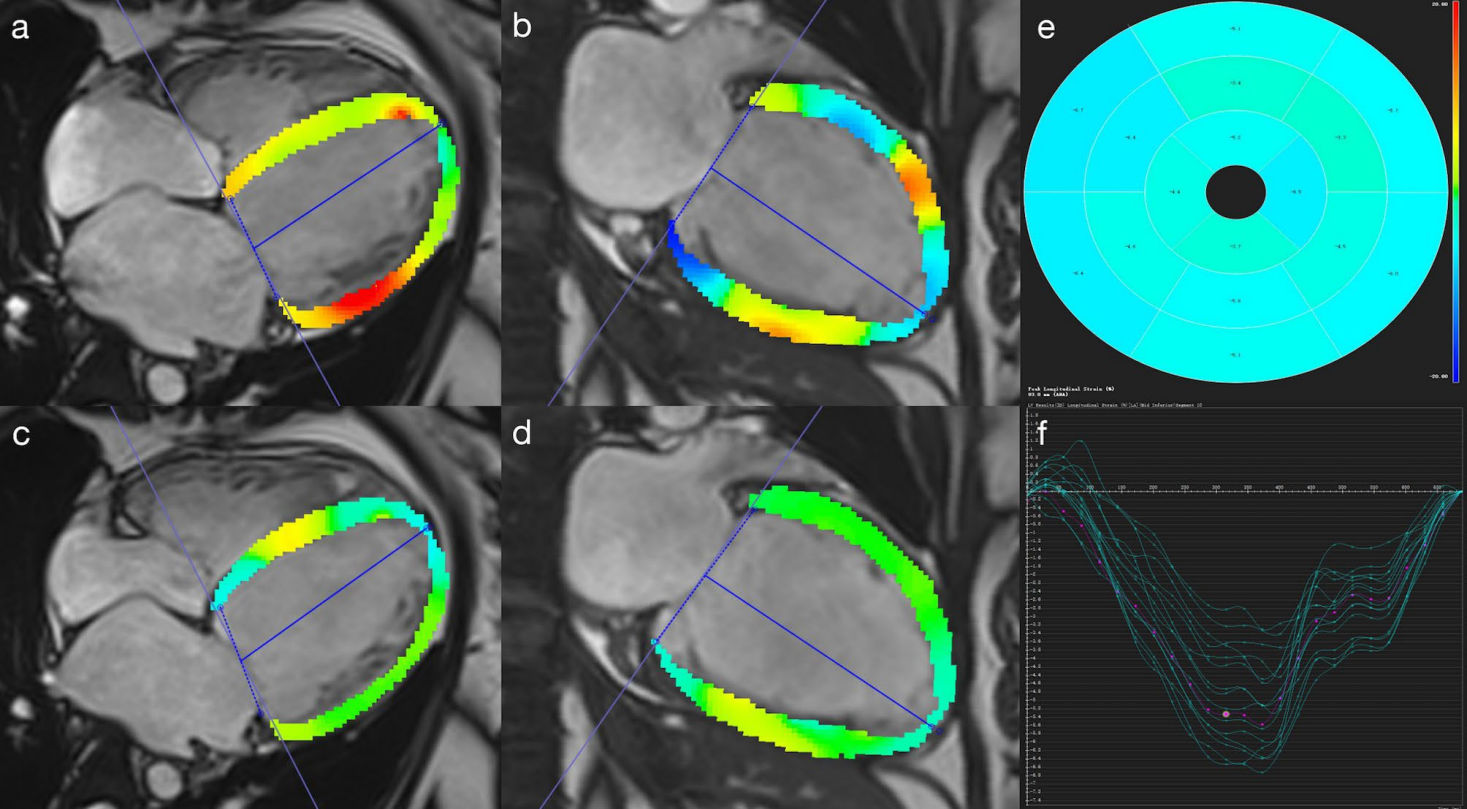
Represent case of global peak longitudinal strain reduction in LVNC combined with MR at the end-systole **(a,b)** and end-diastole **(c,d)**. **(a–d)** General reduction (coloration from light blue to light green) of peak longitudinal strain (absolute value). **(e–f)** The absolute value of global peak longitudinal strain showed significantly reduced.

In all patients with MR, the global and segmental peak radial strain (PRS) and peak circumferential strain (PCS) were lower than patients without MR (all P < 0.05). In LVNC patients, the peak longitudinal strain (PLS) of patients with MR was lower than that of patients without MR, and the difference was statistically significant (Global PLS, − 3.38 ± 4.40 vs − 8.35 ± 3.97; Basal PLS, − 2.85 ± 6.07 vs − 7.73 ± 4.39; Middle PLS, − 3.34 ± 5.09 vs − 7.87 ± 4.01; Apical PLS, − 3.16 ± 5.83 vs − 9.98 ± 4.28, all P < 0.05), the representative images of GPLS reduction in LVNC patients with MR are shown in Fig. 4. In all patients without MR, the global and regional PLS of LVNC patients was higher than that of hypertrabeculation patients (GPLS, − 8.35 ± 3.97 vs − 5.09 ± 6.13; BPLS, − 7.73 ± 4.39 vs − 4.96 ± 4.34; MPLS, − 7.87 ± 4.01 vs − 5.32 ± 4.99; APLS, − 9.98 ± 4.28 vs − 3.89 ± 14.89, P < 0.05), more details are shown in Table 3. In addition, there was no significant difference in strain in patients with different degree of MR (Fig. 3c).

**Table 3:** The global and regional myocardial strain.

	Hyper-MR^−^ N = 53	Hyper-MR^+^ N = 46	LVNC-MR^−^ N = 43	LVNC-MR^+^ N = 40
**Global**				
GPRS	12.86 ± 9.54	7.23 ± 4.17*	16.86 ± 11.01	7.5 ± 4.73^#^
GPCS	− 10.30 ± 5.16	− 6.80 ± 3.30*	− 11.16 ± 4.59	− 6.65 ± 3.2^#^
GPLS	− 5.09 ± 6.13	− 3.58 ± 3.67	− 8.35 ± 3.97*	− 3.38 ± 4.40^#^
**Regional**				
BPRS	21.50 ± 12.64	12.08 ± 7.59*	22.59 ± 13.68	12.64 ± 7.32^#^
BPCS	− 9.59 ± 4.24	− 6.20 ± 2.82*	− 9.74 ± 3.33	− 6.7 ± 2.5^#^
BPLS	− 4.96 ± 4.34	− 2.46 ± 4.55	− 7.73 ± 4.39*	− 2.85 ± 6.07^#^
MPRS	11.60 ± 9.06	6.64 ± 3.95*	16.07 ± 11.47	7.91 ± 6.36^#^
MPCS	− 9.79 ± 5.32	− 6.43 ± 3.17*	− 10.94 ± 4.82	− 6.11 ± 3.03^#^
MPLS	− 5.32 ± 4.99	− 3.58 ± 4.26	− 7.87 ± 4.01*	− 3.34 ± 5.09^#^
APRS	9.84 ± 11.02	5.18 ± 5.41	16.69 ± 17.28*	4.79 ± 5.13^#^
APCS	− 12.11 ± 6.67	− 8.37 ± 5.09*	− 13.45 ± 6.27	− 7.19 ± 5.88^#^
APLS	− 3.89 ± 14.89	− 4.72 ± 4.87	− 9.98 ± 4.28*	− 3.16 ± 5.83^#^

*, vs Hyper-MR^−^; ^#^, vs LVNC-MR^−^.

*GPRS* global peak radial strain, *GPCS* global peak circumferential strain, *GPLS* global peak longitudinal strain, *BPRS* basal peak radial strain, *BPCS* basal peak circumferential strain, *BPLS* basal peak longitudinal strain, *MPRS* middle peak radial strain, *MPCS* middle peak circumferential strain, *MPLS* middle peak longitudinal strain, *APRS* apical peak radial strain, *APCS* apical peak circumferential strain, *APLS* apical peak longitudinal strain.

### Correlation between clinical characteristics and function in LVNC patients with and without MR

In this study, EDV, ESV, and global PLS were statistically associated with mitral regurgitation and NYHA class (P < 0.05), but the NC/C ratio had no significant correlation with mitral regurgitation or NYHA classification. EF, 2D SI, and global PRS were negatively correlated with mitral regurgitation and NYHA class (P < 0.05). 3D SI, MaxMin EDDR, and L-shorten were statistically associated with mitral regurgitation but not with NYHA class.

## Discussion

This study systematically analyzed the prevalence of LVNC combined with MR and the effects of MR on cardiac structure, function, and left ventricular strain in LVNC patients. The main findings are as follows: (1) MR occurred in more than half in LVNC patients with cardiac dysfunction; (2) MR was closely associated with left ventricular geometric remodeling in LVNC patients, especially those with end-diastolic and end-systolic enlargement; (3) MR was associated with an overall decrease in left ventricular strain in LVNC patients but not with the severity of myocardial hypertrabecularization. These observations suggest that MR has an important effect on cardiac morphology and function in LVNC patients, that LVNC with MR may have a worse clinical prognosis, and that it is worth discussing whether to intervene for MR in this subclass of patients.

### Assessment of mitral regurgitation

MR is common in heart disease, and it carries a poor prognosis in populations at high risk for complications^14^. Echocardiography is widely used in the assessment of MR, but CMR shows promise for the assessment of mitral regurgitation and other complementary information^15^. In the assessment of cardiac structure and function, cine CMR depicts good myocardial-blood contrast, and scanning time is relatively short. Moreover, MR can also be assessed semi-quantitatively through evaluation of the signal voids of the regurgitation jet through the cine sequence^10^. Although cine CMR has some deficiencies, this simple and intuitive imaging method may be highly practical for initial assessment. The newly expert consensus has further clarified the clinical utility and value of CMR in the assessment of MR^16^.

### Morphology and function

There is growing evidence in the literature that the presence of MR plays an important role in left ventricular remodeling and dysfunction in cardiomyopathy^7^. Our study further found that among LVNC patients, 2D SI, MaxMin EDDR and L-shorten were significantly lower in patients with MR than in those without MR, suggesting that the presence of MR is closely related to changes in left ventricular geometry in LVNC patients. The reasons may be progressive ventricular reconstruction separates the papillary muscles and changes the natural vertical angle of the chorda tendineae, thereby tethering the leaflets to enhance MR^17^. In addition, the EDV and ESV of the LV increased significantly in patients with mitral regurgitation, but there was no significant correlation with the degree of trabecularization. This result was similar to previous studies showing that a greater extent of LV trabeculation is not associated with an absolute increase in EDV and ESV over almost ten years in normal participants in the MESA (Multi-Ethnic Study of Atherosclerosis) study^18^. There were more studies that thought the presence of excessive trabeculation has no meaningful independent prognostic value in asymptomatic low-risk populations or in cardiomyopathies^19^. However, changes in ventricular volume were more significant in our patients with MR, and some studies have found that heart failure and lethal ventricular arrhythmias may develop if the left ventricular EDV index exceeds 55 ml/m^2^^17^. Our study also found that an increased EDV is directly related to decreased cardiac function, the high incidence of arrhythmia in our cohort with MR also corroborates this result. The consistency of the results suggests that the degree of trabecularization is not the main factor affecting cardiac function, and MR may play an important role in this process.

### Strain and mitral regurgitation in LVNC

CMR cine strain analysis is a good indicator of cardiac motor function. In this study, LVNC patients with MR had significantly lower global and segmental strain than patients without MR. Previous studies found the appearance of MR can significantly affect left ventricular strain in dilated cardiomyopathy, hypertrophic cardiomyopathy, and ischemic cardiomyopathy^20,21^. Our study further found that MR affected strain in LVNC patients, and this phenomenon has not been fully studied at present. Studies have shown that myocardial strain damage is associated with noncompaction and mitral regurgitation, Dreisbach et al. found LVNC patients had lower PLS than healthy subjects, but was less serious than that of dilated cardiomyopathy^20,22,23^, the difference in the arrangement of the trabeculae is probably the main reason^24^. In our study, it was found when LVNC and MR occurred simultaneously, PLS significantly reduced. Longitudinal strain reflects the motion of longitudinal myocardial fibers located in the innermost layer of the myocardium, which is also the main site of LVNC lesions^25^. Impaired PLS experienced higher mortality rates than did those with a more preserved LV PLS^26^.A series of studies have found that a lower LV GLS was independently associated with left ventricular dysfunction and a lower chance of LV reverse remodeling and with higher mortality and cardiac event rates during follow-up^7,26–28^. This highlights that LVNC patients with MR have a low chance of left ventricular retrograde reconstruction and a worse prognosis, so more attention and intervention measures should be directed at these patients clinically.

Some studies suggest that increased severity of mitral regurgitation leads to decreased left ventricular contractile reserve^15,29,30^, and this change may affect the strain of the left ventricle. Although strain changes tend to decrease in patients with severe MR, the interesting finding in the present study was that the degree of MR had no significant correlation with strain parameters. The change in strain was only related to the presence or absence of regurgitation. The reason may be that the severity of MR is related to left ventricular myocardial contractility, left ventricular volume, and cross-valve pressure differences^31^. Most of our participants also had impaired systolic function, and the left ventricular ESV and EDV increased significantly, which may have affected the accuracy of the judgment of the severity of regurgitation. In addition, the presence of reverse flow through mitral valve to the left atrium is a good parameter for MR screening. However, the severity of mitral regurgitation may be underestimated when left atrial pressure is too high or systemic blood pressure is low, and may be overestimated when systemic blood pressure is too high^32^. Nevertheless, these results suggest that the presence of mitral regurgitation, regardless of the degree, may have a more severe effect on the myocardium. It would provide the clinician with diagnostic and prognostic information that can contribute importantly to identifying a subset of LVNC patients at higher risk.

### The prevalence of mitral regurgitation in LVNC

In this study, more than half of LVNC patients had MR, and the prevalence of MR was significantly higher than the 21% previously reported^33^. The reason may be that the patients included in this retrospective study went to hospital due to some extent of cardiac complaint or that the severity of the cardiac lesions may be greater than that of previous studies, causing the secondary MR ratio to be correspondingly increased. In addition, the fact that LVNC patients are more prone to MR may be caused by a complex mechanism, which we suspect may be related to the increase in trabeculae in the myocardium and the function of the accessory structure of the annulus due to complex hemodynamic changes. Current studies have shown that MR is common in LVNC patients, especially those with cardiac insufficiency.

### Clinical perspective

Our study evaluated the clinical significance of mitral regurgitation in cardiac morphology and function in LVNC patients, which is an important supplement to the research on the relationship between mitral regurgitation and cardiomyopathy. The presence of LVNC combined with MR may be significantly alter cardiac function and prognosis. These patients should receive more attention in clinical disease management and treatment. However, according to the current guidelines for evaluating the degree of mitral regurgitation, there is a lack of true gold standard, which method quantifies MR severity with the highest degree of accuracy and reliability is unknown^16^, the accuracy of this semi-quantitative evaluation of regurgitation severity needs further study, the 4D-flow, which produces a direct quantification of MR by quantifying flow directly at the valve and is valid in the presence of multiple valve lesions or shunt flow^34^, to investigate the clinical benefit in quantification regurgitation are warranted.

### Limitations

Our study has some limitations. First, this was a retrospective study, and the results should be interpreted cautiously. Second, we did not follow up with the patients for adverse cardiac events, so the long-term impact of MR on morbidity and mortality was unclear. Finally, we did not include normal controls for comparison; however, in this study, patients without MR who had hypertrabecularization and LVNC were used as the control group, and the results remained significant.

## Conclusion

In summary, MR is common in LVNC patients and significantly associated with left ventricular geometry and functional changes in LVNC. However, in terms of how to improve the prognosis and survival rate of such patients, there are no relevant clinical recommendations at present.

## Data Availability

The datasets used during the current study are available from the corresponding author on reasonable request.
